# The Microbiota in the Diagnosis and Treatment of Autism Spectrum Disorder

**DOI:** 10.3390/ijms27125636

**Published:** 2026-06-22

**Authors:** Ekaterina A. Trifonova, Roman A. Ivanov, Alex V. Kochetov, Sergey A. Lashin

**Affiliations:** 1 Federal Research Center Institute of Cytology and Genetics, Siberian Branch of the Russian Academy of Sciences, 630090 Novosibirsk, Russia; ivanovromanart@bionet.nsc.ru (R.A.I.); ak@bionet.nsc.ru (A.V.K.); lashin@bionet.nsc.ru (S.A.L.); 2 Natural Science Faculty, Novosibirsk National Research State University, 630090 Novosibirsk, Russia

**Keywords:** autism spectrum disorder (ASD), microbiota, microbiota–gut–brain axis, mTOR signaling pathway, fecal microbiota transplantation (FMT), probiotics, ketogenic diet (KD)

## Abstract

Autism spectrum disorder (ASD) is a complex neurodevelopmental condition characterized by impaired social interaction, communication deficits, and repetitive behaviors. Recent research highlights the role of the gut microbiota in ASD pathophysiology, particularly through the microbiota–gut–brain axis. The microbiota may influence neurodevelopment via multiple signaling pathways, including the GABAergic and serotonergic systems, as well as the mTOR signaling pathway. This analytical review examines current evidence on microbiota alterations in ASD and evaluates microbiota-targeted strategies for diagnosis and treatment, focusing on fecal microbiota transplantation (FMT), probiotics, and diet-based therapeutic approaches. The review also provides a critical appraisal of the microbiota–gut–brain axis in the context of autism spectrum disorder.

## 1. Introduction

According to the World Health Organization, ASD affected approximately 1 in 127 worldwide in 2021 [1]. At the same time, the U.S. Centers for Disease Control and Prevention’s ADDM Network estimates that ASD prevalence was approximately 1 in 31 children aged 8 years in 2022, with incidence rates rising annually [2]. The core symptoms of ASD are the early onset of communicative and social problems, including speech communication, imagination, stereotyped behavior, and limited interests.

A notable feature of ASD is the high prevalence of gastrointestinal (GI) symptoms. Up to 69% of children with ASD experience constipation, diarrhea, abdominal pain, or other GI disturbances, with an estimated overall prevalence of approximately 33% [3]. At the same time, emerging evidence indicates that gut microbiota profiles and metabolic activity in children with ASD differ markedly from those in their neurotypical peers [4]. Such disparities may contribute to conditions like increased intestinal permeability (“leaky gut”) and immune dysregulation, while also leading to the production of microbial metabolites that can influence the central nervous system [5]. This suggests that the gut microbiome could be both a factor in the pathogenesis of ASD and a modifiable target for reducing symptom severity.

For this review, a flexible search was conducted in February 2026 by means of PubMed, using a search filter for studies written in English. The search terms were as follows: (ASD OR autistic* OR autism*) AND (microbiota* OR microbiome* OR bacteria*); (ASD OR autistic* OR autism*) AND (probiotic*); (ASD OR autistic* OR autism*) AND (diet*). Additionally, names of some bacterial species such as Akkermansia muciniphila and Lactobacillus reuteri were used as search terms. Only studies published no earlier than 2010 were selected for analysis.

Focusing on the microbiota–gut–brain (MGB) axis in the context of ASD, this analytical review proceeds in several key stages: first, it outlines the principal communication pathways and mechanisms; second, it examines the evidence for alterations in the microbiota among individuals with ASD; and finally, it discusses both established and prospective therapeutic approaches, including probiotics, fecal microbiota transplantation, and nutritional interventions.

## 2. The Microbiota–Gut–Brain Axis: Pathways and Mechanisms

**Figure 1:**
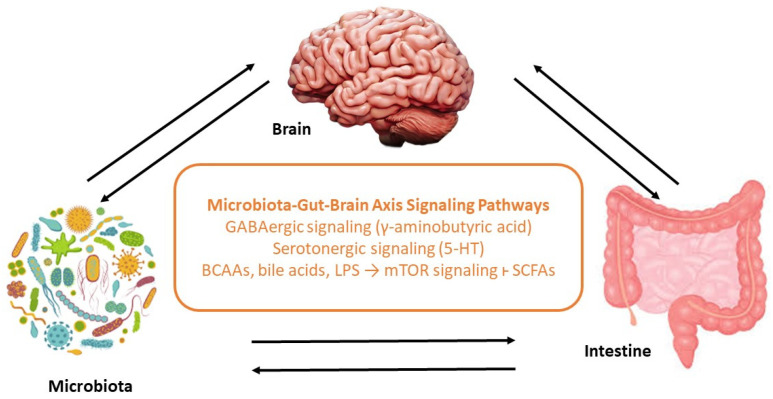
Bidirectional communication of the microbiota–gut–brain axis. The brain, gut, and microbiome form a three-node bidirectional network. 5-HT, serotonin; SCFAs, short-chain fatty acids; BCAAs, branched-chain amino acids; LPS, lipopolysaccharide, →, activation; ⱶ, inhibition.

The microbiota–gut–brain axis is a bidirectional communication system involving neural, endocrine, metabolic, and immune signaling. Anatomically, the vagus nerve primarily provides a direct connection between the intestine and the brain throughout life. Microbiota could affect neurodevelopment in many interrelated ways, in particular, through the synthesis of neuroactive compounds. Intestinal microorganisms synthesize neurotransmitters, including γ-aminobutyric acid (GABA) and serotonin (5-HT), as well as metabolites such as short-chain fatty acids (SCFAs), which can modulate brain function either directly or indirectly [6,7] (Figure 1).

Nearly 90% of serotonin is synthesized in the intestine from tryptophan, and central serotonin deficiency accompanied by peripheral hyperserotonemia has been reported in ASD, probably reflecting impaired serotonergic signaling associated with behavioral deficits [8].

The inhibitory neurotransmitter GABA, which plays a crucial role in neural regulation, is synthesized by specific gut bacteria (including *Bacteroides*, *Bifidobacterium*, and *Lactobacillus*) through the fermentation of glutamate. Alterations in the population size or functionality of these bacteria could lead to decreased GABA availability in the central nervous system (CNS) [9] (Figure 1). Dysregulation of GABAergic signaling is associated with the excitatory-inhibitory imbalance seen in ASD, a mechanism that might contribute to neuronal hyperexcitability, anxiety symptoms, and repetitive behaviors [10].

While the microbiota–gut–brain axis has been commonly linked to GABAergic and serotonergic signaling, the role of mTOR signaling has received less attention. Meanwhile, mTORC1 deregulation has repeatedly been proposed as a common pathological mechanism in ASD [11,12] (Figure 1).

The mTOR pathway exists in two functionally distinct complexes: mTOR complex 1 (mTORC1) and mTOR complex 2 (mTORC2) [13]. mTORC1, sensitive to rapamycin, integrates inputs from growth factors, energy status, oxygen levels, and nutrient availability (especially that of amino acids) to regulate protein synthesis, autophagy, and metabolism. mTORC2, largely insensitive to acute rapamycin treatment, primarily regulates the actin cytoskeleton and cell survival via Akt phosphorylation [13].

The microbiota influences mTOR signaling through multiple interconnected mechanisms. Microbial fermentation produces short-chain fatty acids (SCFAs), including butyrate, propionate, and acetate, which play distinct roles in modulating mTOR activity. For instance, butyrate has been shown to inhibit mTORC1 in intestinal epithelial cells, thereby promoting autophagy and enhancing gut barrier integrity [14]. Meanwhile, propionate has been implicated in neurodevelopmental processes: Sharma et al. [15] highlighted its significant contribution to the development of autism spectrum disorder (ASD) by modulating key molecular pathways—including mTOR and cytokine-activated pathways—during critical prenatal and neonatal periods.

In contrast to the inhibitory effects of certain SCFAs, branched-chain amino acids (BCAAs), produced by lactic acid bacteria (LAB) such as *Lactobacillus* and *Staphylococcus* species, can activate mTORC1 [16]. Furthermore, through the microbiota–gut–brain axis, SCFAs and other microbial metabolites are capable of modulating mTORC1 activity in neurons and glial cells. This modulation has downstream effects on neurodevelopment and behavior [17].

The gut microbiota facilitates the conversion of primary bile acids into their secondary derivatives, including deoxycholic acid. These modified bile acids function as signaling molecules that interact with receptors like FXR and TGR5. Through these receptor-mediated pathways, they can influence mTOR activity in tissues such as the liver and intestine [18].

Additionally, microbial pathogen-associated molecular patterns (PAMPs), such as lipopolysaccharide (LPS), interact with pattern recognition receptors (PRRs) on host cells. This interaction triggers the inflammatory NF-κB pathway, which subsequently induces the expression of chemokine (C-C motif) ligand 5 (CCL5) and leads to the activation of mTOR signaling. This mechanism illustrates how microbial components can directly influence host cell signaling and immune responses via mTOR [19] (Figure 1).

## 3. Microbiota Alterations in ASD

In children with ASD, alterations in gut microbiota composition and function are often observed before age 3, coinciding with the onset of behavioral deficits [20]. Recent systematic and multi-kingdom studies have identified consistent patterns of gut microbiome dysbiosis in ASD. Below is a detailed synthesis of key findings from the latest research.

Patients with ASD have consistently shown reduced alpha diversity (within-subject microbial diversity) compared with neurotypical controls [21,22]. This reduction in diversity has been associated with a less resilient and more unstable gut ecosystem, potentially influencing metabolic and immune functions.

At the phylum level, an increased *Firmicutes* to *Bacteroidetes* ratio has been numerously reported in ASD, which has often been linked to inflammation and metabolic disturbances [23,24]. In particular, a lower relative abundance of *Bacteroidetes* has been observed in children with more severe ASD symptoms [22,25].

*Proteobacteria* have shown mixed results: most studies have reported higher levels in ASD [26,27,28], but there was also an opposite finding [29]. Similarly, *Actinobacteria* have been generally more abundant in ASD [26,28,30], although a decrease has been reported as well [31].

Several genera have been consistently reported as more abundant in ASD: 1. *Desulfovibrio* spp., a sulfate-reducing bacteria linked to gut barrier dysfunction [24,32]; 2. *Sutterella* spp., implicated in mucosal inflammation [33,34]; 3. *Candida* spp. (fungal genus), potentially contributing to immune activation [35,36]; 4. *Clostridium* spp. (especially clusters I and XI), associated with elevated production of p-cresol and other phenolic compounds, which may cross the blood–brain barrier and affect neurodevelopment [21,37,38].

Meanwhile, *Bifidobacterium* spp., known for anti-inflammatory and intestinal barrier supporting roles [39,40], and *Akkermansia muciniphila*, a mucin degrader associated with gut barrier integrity [40,41], have been reported to be less abundant in ASD [21] (Table 1).

Together, these alterations may contribute to both gastrointestinal distress and core autism symptoms through inflammatory and neuroactive pathways. However, these observations should be interpreted cautiously: most studies are cross-sectional, and taxonomic differences alone do not establish whether microbiota changes precede ASD-related symptoms, result from diet and gastrointestinal comorbidities, or reflect other environmental and clinical confounders. Current research into gut microbiota in ASD has primarily relied on fecal samples, which reflect the composition of the colon microbiota but may not capture changes in other gut regions. According to a systematic review by Korteniemi et al. [21], 49 out of 51 studies included in the analysis used fecal microbiota samples for sequencing. This highlights a critical gap: the small intestinal microbiota has remained largely unexplored in ASD research, despite its potential role in immune modulation and nutrient absorption.

The distinction between small intestine and colon microbiomes is crucial. As Jensen et al. [44] emphasized, these two regions differ significantly in microbial composition; thus, *Streptococcaceae* have been predominantly found in the small intestine, participating in early carbohydrate metabolism. In addition, key immune cells and lymphoid tissues (e.g., Peyer’s patches) are concentrated in the small intestine, which is essential for microbiota–immune–brain crosstalk. Finally, the small intestine has faster transit times and different pH levels compared with the colon, shaping distinct microbial niches [44]. These differences have raised questions about whether ASD-associated dysbiosis has originated in specific gut regions and whether fecal samples alone could provide a complete picture of gut–brain interactions.

Until recently, the oral microbiome, which is more accessible for sampling, had not been systematically studied in ASD. However, Manghi et al. [43] performed a large-scale, multi-cohort metagenomic analysis of the oral microbiome and identified microbial taxa and functional pathways associated with ASD. Significant differences in oral microbiome composition were observed between ASD and control groups, including increased abundance of *Streptococcus mutans*, *Prevotella melaninogenica*, *Fusobacterium nucleatum*, and *Veillonella parvula* and decreased abundance of *Rothia mucilaginosa*, *Neisseria flavescens*, and *Haemophilus parainfluenzae*. Microbial metabolic pathways were also differentially represented with enrichment in pathways related to LPS biosynthesis, amino acid fermentation, neuroactive metabolite production (e.g., GABA precursors), and depletion in pathways involved in carbohydrate metabolism and SCFA production.

A strong correlation has been found between the relative abundance of microbial taxa and cognitive impairment (measured by Full-Scale IQ). Among children with ASD, those with an IQ < 70 have shown markedly lower oral microbiome strain sharing with parents (*p* < 10^−6^) relative to neurotypical controls. Sex-specific patterns have been minimal, with no significant interaction between sex and microbiome composition in ASD. However, gastrointestinal comorbidities in ASD have been associated with more pronounced shifts in microbial profiles, particularly with increased abundances of *Fusobacterium* and *Veillonella* [43].

## 4. Microbiota as a Candidate Diagnostic Tool

Emerging data suggest that microbial profiles, particularly those of the gut and oral microbiomes, could serve as non-invasive, early-life biomarkers for autism spectrum disorder. This approach is also closely related to the growing understanding of the microbiota–gut–brain axis and its role in neurodevelopment.

A large-scale metagenomic study revealed that the oral microbiome contains robust and generalizable microbial and functional signatures associated with ASD [43]. It was demonstrated that the composition of the oral microbiome could effectively discriminate individuals with ASD from their NT siblings, achieving an area under the curve (AUC) of 0.66. The analysis identified 108 bacterial species with statistically significant differences between the groups (q < 0.005). Functional enrichment analysis revealed that enzymes from serotonin, GABA, and dopamine degradation pathways shape the distinct microbial communities found in ASD versus NT samples [43]. These findings have supported further research into non-invasive microbiome-based screening tools and have provided new insights into possible microbiome-neurodevelopment links.

The diagnostic potential of gut microbiota profiling in ASD has been limited by the reliance on fecal samples. However, emerging methods may allow non-invasive assessment of small intestinal microbiota, addressing a key gap we have identified in Korteniemi et al.’s review [21].

To complement fecal sequencing approaches, analytical methods based on microbial markers have been proposed by Osipov and Verkhovtseva for indirect assessment of mucosa-associated and small intestinal microbiota [45]. This method detects microbial chemical signatures (e.g., fatty acids, alcohols) in blood or urine, potentially reflecting near-wall small intestinal microbial activity without invasive sampling. This approach has several potential advantages, including rapid analysis, culture independence, and applicability to non-fecal samples. At the same time, its taxonomic interpretation depends on the specificity and completeness of reference marker libraries. More broadly, lipid- and fatty-acid-based community profiling should be interpreted cautiously when inferring detailed microbial biomass or taxonomic composition, because some taxa may lack distinctive chemical signatures, marker yields may differ between taxa, and fatty acid profiles can be influenced by biological and experimental conditions. Our pilot study involving mass-spectrometry-based profiling of microbial markers in 100 peripheral blood samples obtained from the Russian ASD patient population has demonstrated markedly elevated levels of *Streptococcus* spp., *Clostridium* spp., *Propionibacterium acnes*, and *Actinomyces* spp., coupled with reduced abundances of *Prevotella* spp. and *Fusobacterium* spp. [42] (Supplementary Figure S1). If validated in ASD populations, this technique could enhance diagnostic accuracy by integrating data from multiple gut regions.

## 5. Microbiota-Targeted Treatments

### 5.1. Fecal Microbiota Transplantation

Fecal Microbiota Transplantation (FMT) involves transferring gut microbiota from a healthy donor to a recipient in order to restore microbial balance. A landmark, open-label study investigated the efficacy of FMT for improving gastrointestinal (GI) and ASD symptoms [46]. Fecal microbiota transplantation—consisting of 2 weeks of antibiotics, bowel cleansing, and extended FMT (initial high dose followed by 7–8 weeks of daily maintenance doses)—reduced gastrointestinal (GI) symptoms by ~80%, as measured by the Gastrointestinal Symptom Rating Scale. Improvements in constipation, diarrhea, indigestion, and abdominal pain persisted 8 weeks post-treatment.

ASD-related behavioral symptoms have also shown a significant and sustained improvement over the same period. Bacterial and phage sequencing analyses revealed evidence of partial donor microbiota engraftment, increased bacterial diversity, and higher abundances of *Bifidobacterium*, *Prevotella*, *Desulfovibrio*, and other taxa; these changes persisted for 8 weeks after FMT completion. However, because the study was open-label, lacked blinded placebo-controlled assessment, and included several simultaneous intervention components, these behavioral findings should be interpreted cautiously, especially where outcomes depend on parent or caregiver ratings [46].

Another open-label study [47] demonstrated that FMT promoted donor microbe colonization and shifted the gut bacterial profile of children with ASD towards that of NT controls. Pre-FMT, higher levels of *Eubacterium coprostanoligenes* correlated with worse gastrointestinal symptoms (higher GSRS scores), and its FMT-induced reduction was linked to treatment response. This taxon may therefore represent a candidate microbial correlate of gastrointestinal symptom change, although its mechanistic role and clinical relevance require further validation [47].

Although preliminary observational studies have reported a notable symptom improvement in children with ASD following FMT compared with baseline levels, the safety and efficacy of FMT as a therapeutic intervention require validation through rigorously designed randomized controlled trials with blinded assessment, predefined endpoints, appropriate placebo/sham controls where ethically feasible, and long-term follow-up [48]. Safety concerns are particularly important. FMT can transmit bacteria, viruses, and other organisms; regulatory safety communications have reported serious infections, including multidrug-resistant organisms and pathogenic *Escherichia coli*, after investigational FMT [49,50].

### 5.2. Probiotics

Probiotics are non-pathogenic living microorganisms that may confer health benefits to the host when consumed in adequate quantities as part of the diet or as dietary supplements. Probiotic interventions in the trials predominantly featured strains from the *Lactobacillus* and *Bifidobacterium* genera, including *L. plantarum* [51], *L. acidophilus* [52], a three-strain mix (*L. acidophilus*, *L. rhamnosus*, *B. longum*) [53], and a five-strain combination (*L. acidophilus*, *L. casei*, *L. delbrueckii*, *B. longum*, *B. bifidum*) [54]. Few studies have examined how probiotics affect ASD core and emotional symptoms. Among those that have, *Lactobacillus acidophilus*, *L. plantarum*, *L. rhamnosus*, and *Bifidobacterium longum* have shown promising results in reducing ASD severity, but these findings are strain-specific, heterogeneous, and often based on small samples or open-label designs [52,53,55].

*Limosilactobacillus reuteri* (or *Lactobacillus reuteri*) is a recently introduced probiotic. A first-in-human, single-center, randomized, placebo-controlled, double-blind, crossover trial was conducted to evaluate the safety and efficacy of SB-121 (*Limosilactobacillus reuteri* (*L. reuteri*), Sephadex^®^ (dextran microparticles), maltose) in 15 participants with autism [54]. The placebo-controlled, double-blind, crossover trial involved daily dosing for 28 days, followed by a 14-day washout period and another 28-day phase with the alternative treatment (SB-121 or placebo).

The results showed that SB-121 was safe and well tolerated over the short trial period: adverse event rates were similar between the SB-121 and placebo groups, with most events reported as mild. Moreover, the study revealed promising clinical signals. The participants demonstrated a statistically significant improvement in adaptive behavior, as measured by the Vineland-3 Adaptive Behavior Composite score (*p* = 0.03), during SB-121 treatment. Additionally, there was a trend towards an increased social/geometric viewing ratio, indicating enhanced social preference, when assessed via eye-tracking methods compared with placebo. These findings support further clinical evaluation of SB-121, but the small sample size and short duration preclude firm conclusions about efficacy for ASD [56].

In a pilot single-arm study, 17 children with ASD received *Lactobacillus reuteri* LR-99 (5.0 × 10^10^ CFU, thrice daily) for 4 weeks. The intervention led to a significant reduction in gastrointestinal symptoms (lower GSRS scores, improved stool consistency on BSFS), improvement in core autistic behaviors (lower CARS and SRS scores), and a shift in gut microbiota profile towards that of NT controls. These results indicate that *L. reuteri* LR-99 supplementation may alleviate gastrointestinal and behavioral symptoms while modulating the gut microbiota in children with ASD [57]. Overall, probiotic data in ASD remain heterogeneous. Small samples, different strains and doses, short treatment periods, and reliance on behavioral rating scales limit conclusions about core ASD symptoms. Research into probiotics for ASD would be more informative if directed towards clinically defined subgroups, especially children with gastrointestinal comorbidities, instead of being framed as a broadly applicable treatment.

### 5.3. Dietary Interventions

Diets such as gluten-free/casein-free (GFCF) have been explored for their impact on microbiota and ASD symptoms. A 24-month randomized controlled trial investigated the effects of a gluten- and casein-free diet in children with ASD [58]. The two-stage study with an adaptive ‘catch-up’ design involved 72 Danish children aged 4 to almost 11 years. In Stage 1, the participants were stratified and randomly assigned to either a dietary intervention group (A) or a non-diet control group (B).

Core ASD-related behaviors were assessed using the Autism Diagnostic Observation Schedule (ADOS) and the Gilliam Autism Rating Scale (GARS), developmental levels were assessed via the Vineland Adaptive Behavior Scales (VABS), and symptoms of inattention and hyperactivity were assessed with the ADHD-IV scale. Evaluations took place at baseline and at 8 and 12 months. At the 12-month mark, per-protocol analysis of data from 26 children in the diet group and 29 controls revealed significant improvements in the diet group. Specifically, mean scores showed positive changes in sub-domains measured by ADOS, GARS and ADHD-IV, driven by a significant time-by-treatment interaction. These results exceeded predefined statistical thresholds, prompting the re-assignment of the control group (B) to the active dietary intervention for Stage 2.

In Stage 2, data from 18 participants originally assigned to group A and 17 from group B were available at the 24-month endpoint. Multiple scenario analyses, including inter- and intra-group comparisons, indicated sustained clinical improvements, though the signs of a plateau effect emerged, suggesting that the intervention’s impact might level off over time.

The findings suggest that a gluten- and casein-free diet may positively influence the developmental outcomes for some children with ASD. However, the lack of a placebo condition and the difficulty of blinding dietary interventions mean that expectancy effects and non-dietary aspects of the intervention cannot be ruled out. Further research is needed to identify which children are most likely to benefit from this dietary approach and which ones may not respond or may be harmed by unnecessary restriction [58].

The ketogenic diet (KD), known to alter the gut microbiome and raise blood ketone levels (including β-hydroxybutyrate), has been studied as a possible intervention for improving sociability and other outcomes for some children with ASD. Since ketone bodies can cross the blood–brain barrier, they may provide neuroprotective benefits.

An interventional pilot study [59] investigated the effects of a modified KD on the gut microbiome, inflammatory markers, and brain-related microRNAs (miRNAs) in children with ASD. A 4-month follow-up revealed that KD lowered the plasma levels of proinflammatory cytokines (IL-12p70, IL-1β) and BDNF. It has also induced gut microbiome changes, boosted butyrate kinase expression, and altered BDNF-related miRNA levels in plasma. These findings support the role of reduced inflammation and improved gut microbiota in enhancing sociability in ASD, but the pilot design does not establish causality or general clinical efficacy [59].

A systematic review was conducted that included 13 studies investigating the effects of KD on the gut microbiome in patients with neurological disorders such as epilepsy, autism spectrum disorder, multiple sclerosis, depression, Parkinson’s disease, and Alzheimer’s disease [60]. The authors concluded that after KD, an increase was observed in the abundance of members of the *Proteobacteria* phylum, as well as the genera *Escherichia*, *Bacteroides*, *Prevotella*, *Faecalibacterium*, *Lachnospira*, *Agaricus*, and *Mrakia*. At the same time, there was a decrease in the proportion of the phyla *Firmicutes* and *Actinobacteria*, as well as the genera *Eubacterium*, *Cronobacter*, Saccharomyces, *Claviceps*, *Akkermansia*, and *Dialister*. Following KD, a reduction in the concentrations of fecal short-chain fatty acids (SCFAs) and branched-chain fatty acids was recorded in stool samples, along with increased levels of beta-hydroxybutyrate, trimethylamine N-oxide, and N-acetylserotonin. Therefore, KD prescribed to patients with neurological disorders effectively altered the composition of the gut microbiome and the metabolites derived from it [60].

However, restrictive diets have non-trivial safety considerations in children. Gluten-free/casein-free diets may reduce intake of fiber, calcium, iron, folate, B-group vitamins, zinc, and other micronutrients if they are not carefully planned, and may further narrow an already selective diet in some children with ASD [61]. KD requires medical and dietitian supervision with laboratory and growth monitoring, because adverse effects can include constipation, vomiting or reflux, dehydration, hypoglycemia, metabolic acidosis, dyslipidemia, nephrolithiasis, micro-nutrient deficiency, and impaired growth [62]. Thus, these diets could not be used as universally ASD treatments; they should be considered only with careful nutritional assessment and follow-up.

## 6. Critical Appraisal of the Microbiota–Gut–Brain Axis in Autism Spectrum Disorder

One of the key methodological problems in the literature on the role of the microbiota–gut–brain axis in ASD is the distinction between association and causality [63]. In the review by Cryan and Mazmanian, it was emphasized that although the gut microbiota is capable of influencing brain activity and behavior, the causal role of these processes in humans remains unestablished [64,65]. This point was later developed in a number of methodological reviews [66,67]: numerous correlations between microbiota characteristics and neuropsychiatric features were identified, yet the reproducibility of these associations remains low, and the transition from correlational observations to causal conclusions has been, in most cases, methodologically unjustified. In the context of ASD, this problem is especially important due to the phenotypic heterogeneity of the disorder, the high prevalence of co-occurring gastrointestinal symptoms, the specific features of eating behavior, and the marked sensitivity of the microbiome to environmental influences. This point has been reiterated especially often in the review literature on ASD since most available studies have a cross-sectional or retrospective design [66,67,68]. Such studies could show that, in some children with ASD, the microbiota has differed from that of control groups, but they could not determine whether these changes preceded symptom development, resulted from dietary and behavioral characteristics, or reflected co-occurring gastrointestinal disorders.

A separate body of publications has focused on the role of confounding factors, most notably diet, medication exposure, and the family environment [69,70,71]. Children with ASD have often been characterized by food selectivity, a limited range of products consumed, sensory sensitivity to taste and food texture, and a high prevalence of constipation, as well as other functional gastrointestinal disorders [72,73,74,75]. Each of these factors alone could substantially influence the composition of the gut microbiota. Particularly important in this regard was the study by Yap et al. [76], which proposed a model in which ASD-related restricted interests were associated with a less diverse diet, which, in turn, was linked to reduced taxonomic diversity of the microbiota; Özcan and Hsiao supported a similar interpretation in the commentary [77]. A recent family-based study by Di Benedetto et al. [78] likewise concluded that, in children with ASD, behavioral differences could be better explained by dietary patterns than by microbiome profiles. These findings underscore the need for careful consideration of dietary variability in microbiome research.

Another limitation under discussion is that the overwhelming majority of ASD studies have been based on the analysis of stool samples. A 2023 review devoted to the intestinal barrier and the microbiota–gut–brain axis in ASD explicitly noted that fecal microbiota reflect the contents of the large intestine relatively well, but it is a much less precise indicator of microbial communities in other parts of the gastrointestinal tract [5]. The same review emphasized that, despite the convenience and noninvasiveness of stool-based studies, they could not allow a full assessment of the mucosal microbiota, particularly that of the small intestine, where processes related to immune regulation, digestion, and barrier function may take place [79,80]. The authors also pointed out that the results of biopsy-based studies could not always coincide with fecal findings: for example, analyses of biopsies from the duodenum and ileocecal region revealed features that have not been consistently reproduced in stool-based studies [33,81]. Accordingly, the recent literature has increasingly emphasized that the predominance of fecal studies limits the completeness of conclusions regarding the role of the microbiota in ASD [82]. A more detailed discussion of this problem and possible ways to address it has been provided in the previous chapter of our review.

We are deeply convinced that particular attention should also be paid to moving away from the search for a “general autism biomarker” and toward a stratified model. Given the heterogeneity of ASD, a more realistic goal might be the identification of biomarkers for clinically meaningful subgroups. For example, the similarities in neuropsychological symptoms between syndromic and idiopathic forms of ASD have allowed hypothesizing mTORC1 dysregulation as a common pathological mechanism for at least one subtype of the disorder [11,12]. The percentage of autism spectrum disorders with hypo- and hyperactivated mTOR signaling is still unknown. Certain syndromic forms of ASD, including PTEN hamartoma tumor syndrome (PHTS) and tuberous sclerosis complex (associated with TSC1/TSC2 mutations), are characterized by elevated mTORC1 activity [83]. In contrast, Rett syndrome is associated with globally reduced transcription as well as decreased AKT/mTOR signaling [84]. The ketogenic diet, which inhibits mTOR activity and helps manage metabolic and inflammatory conditions [85], could be a potential therapeutic option for the mTOR-hyperactivated subtype of autism—provided it is implemented with close medical oversight. However, it should be avoided in individuals exhibiting reduced mTOR activity. Such a strategy might improve both the statistical power of studies and the clinical interpretability of their findings.

## Figures and Tables

**Table 1:** Taxa-level abundances in patients with ASD compared with NT controls when more than one study had significant results.

Taxa	Increased in Patients with ASD Compared with NT	Decreased in Patients with ASD Compared with NT
*Firmicutes*/*Bacteroidetes*	↑ ^1^ [23,24]	-
*Proteobacteria*	↑ [26,27,28]	↓ ^2^ [29]
*Actinobacteria*	↑ [26,28,30]	↓ [31]
*Desulfovibrio* spp.	↑ [24,32]	-
*Sutterella* spp.	↑ [33,34]	-
*Candida* spp.	↑ [35,36,42]	-
*Clostridium* spp.	↑ [21,37,38,42]	-
*Bifidobacterium* spp.	-	↓ [39,40]
*Akkermansia muciniphila*	-	↓ [21,40]
*Streptococcus* spp.	↑ [42,43]	-
*Prevotella* spp.	↑ [43]	↓ [42]

^1^ ↑ - increased abundance. ^2^ ↓ - decreased abundance.

## Data Availability

No new data were created or analyzed in this study. Data sharing is not applicable to this article.

## Supplementary Materials

The following supporting information can be downloaded at: https://www.mdpi.com/article/10.3390/ijms27125636/s1.

## Abbreviations

The following abbreviations are used in this manuscript:

ASD	Autism spectrum disorder
mTOR	mTOR signaling pathway
SCFAs	Short-chain fatty acids
FMT	Fecal Microbiota Transplantation
GFCF	Gluten-free/casein-free diet
KD	Ketogenic diet
