# Supplementary figures and images for: The Microbiota in the Diagnosis and Treatment of Autism Spectrum Disorder
Source: Int J Mol Sci. 2026 Jun 22;27(12):5636. doi: 10.3390/ijms27125636 (PMC13300348; doi:10.3390/ijms27125636)

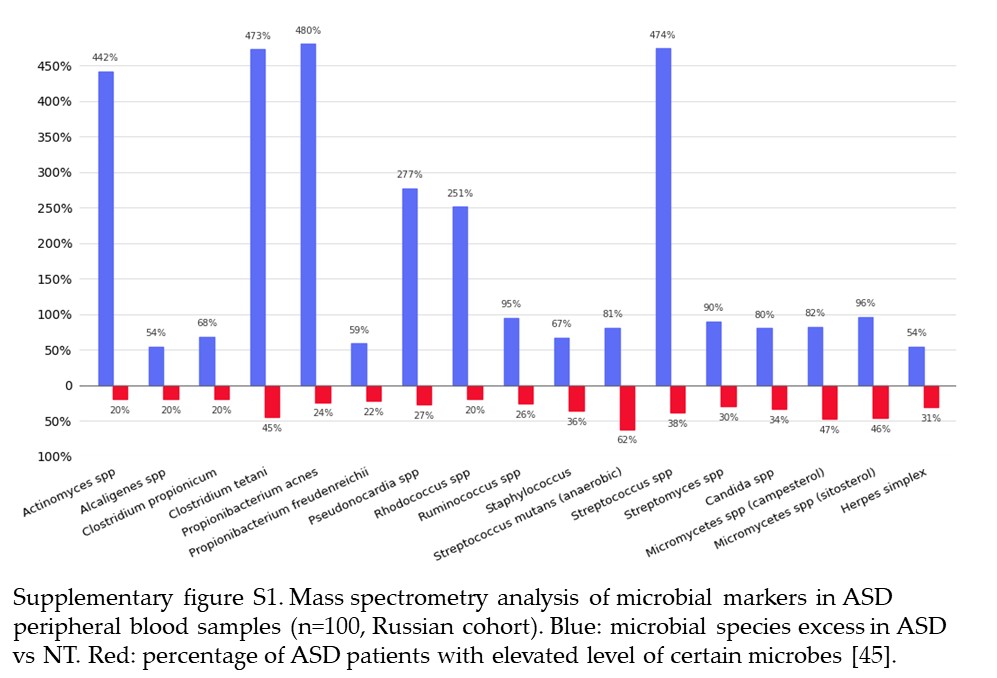

Supplement: Supplementary file 1 [file ijms-27-05636-s001.zip › Supplementary figure S1.jpg]
